# 上皮间质转化在非小细胞肺癌EGFR-TKIs耐药中的研究进展

**DOI:** 10.3779/j.issn.1009-3419.2018.12.08

**Published:** 2018-12-20

**Authors:** 莉 虞, 沙 黄, 望 吕, 哲浩 何, 坚 胡

**Affiliations:** 310003 杭州，浙江大学医学院附属第一医院胸外科 Department of Thoracic Surgery, the First Affiliated Hospital, School of Medicine, Zhejiang University, Hangzhou 310003, China

**Keywords:** 肺肿瘤, EGFR-TKIs耐药, 上皮间质转化, 调控机制, Lung neoplasms, EGFR-TKIs resistance, Epithelial-mesenchymal transition, Regulatory mechanism

## Abstract

肺癌是全世界范围内发病率和死亡率最高的恶性肿瘤之一，其中大约80%是非小细胞肺癌（non-small cell lung cancer, NSCLC）。目前分子靶向治疗已成为NSCLC的一线治疗方法，其中表皮生长因子受体-酪氨酸激酶抑制剂（epidermal growth factor receptor-tyrosine kinase inhibitors, EGFR-TKIs）越来越多地应用于临床治疗，但EGFR-TKI的获得性耐药成为制约EGFR-TKI继续使用的瓶颈。上皮间质转化（epithelial-mesenchymal transition, EMT）是上皮细胞转化为间质表型细胞的生物学现象，可促进肺癌转移、侵袭以及肿瘤细胞获得干性。此外，EMT也是引起NSCLC对EGFR-TKIs耐药的原因之一。有研究发现，逆转NSCLC细胞的间质表型，耐药的细胞能恢复对吉非替尼的敏感性，提示靶向EMT的治疗，或能阻止甚至是逆转NSCLC细胞对EGFR-TKIs的耐药，本文对EMT在NSCLC EGFR-TKIs耐药中的研究进展作一综述。

## Abstract (en)

Lung cancer is the one of the malignant tumor of the highest morbidity and mortality over the world, and non-small cell lung cancer (NSCLC) makes up about 80%. Nowadays, molecular targeted therapy has been the first-line treatment for NSCLC. Epidermal growth factor receptor-tyrosine kinase inhibitors (EGFR-TKIs) are increasingly used in the clinical treatment, but the EGFR-TKIs acquired resistance becomes the bottleneck of continuation of EGFR-TKIs therapy. Epithelial-mesenchymal transition (EMT) is a biological phenomenon in which epithelial cells are transformed into mesenchymal cells. EMT promoted metastasis, invasion of lung cancer and conferred characteristic of stem cell on cancer cells. Meanwhile, EMT is one of an important cause of EGFR-TKIs resistance in NSCLC. The recent studies have found that resistant cells restored the sensitivity to EGFR-TKIs by reversing EMT which suggested that the target of EMT may contribute to inhibit or even reverse the resistance of EGFR-TKIs. Here we make a review about research progress of EMT in EGFR-TKIs resistance in NSCLC.

据统计，我国肺癌发病率每年增长26.9%，肺癌已成为我国首位恶性肿瘤死亡原因，2015年流行病学调查研究结果预测2015年中国新发肺癌数及死亡数分别为733.3/千人和610.2/千人，其中有80%的病例为非小细胞肺癌（non-small cell lung cancer, NSCLC）^[1]^。近年来，随着以表皮生长因子受体（epidermal growth factor receptor, EGFR）为代表的NSCLC驱动基因突变的研究进展，分子靶向药物得到广泛的应用。19外显子缺失、21外显子点突变（L858R）是目前已知*EGFR*最常见的突变类型，且和患者的临床特点有一定的相关性，多见于腺癌、亚洲人种、女性以及不吸烟人群。而这类基因突变患者对第一代EGFR酪氨酸激酶抑制剂（EGFR-tyrosine kinase inhibitors, EGFR-TKIs）如吉非替尼和厄洛替尼，往往是敏感的。其有效率可高达80%，无进展生存期显著延长^[2]^，但有一部分*EGFR*突变患者对EGFR-TKIs原发耐药，且即使对其敏感的患者在治疗过程中也不可避免地产生耐药，有关耐药机制的研究主要集中在T790M、*KRAS*突变，*c-Met*原癌基因扩增等方面。近来研究发现，EGFR-TKIs耐药与肿瘤细胞发生上皮间质转化（epithelial-mesenchymal transition, EMT）密切相关，目前已成为NSCLC EGFR-TKIs耐药研究领域中的一大热点。

## 1 EGFR-TKIs耐药相关机制

目前，EGFR-TKIs耐药机制尚未完全阐明，研究主要集中在以下几个方面：①*EGFR*基因再突变，其中约50%的患者都是由于*EGFR* T790M突变引起的耐药，目前有“亚克隆”和“诱导突变”这两大学说来解释*EGFR*基因T790M再突变现象，此外，D761Y、L747S和T854A等非T790M再突变也会使NSCLC对EGFR-TKIs的治疗敏感性下降；②*K-RAS*突变，K-RAS是一种GTP酶，参与一系列细胞重要活动，而*K-RAS*中第2外显子区域的12和13密码子突变可持续激活RAS，进而诱发肿瘤对EGFR-TKIs的耐药，其中80%的突变都发生在12密码子；③*c-Met*扩增，c-Met是一类受体酪氨酸激酶，参与TK结构域激活以及增殖、迁移、侵袭等各类细胞活动的活化过程，正常生理调控下在稳态维持方面可起到重要作用，而*c-Met*原癌基因的扩增可导致下游PI3K/AKT通路的异常活化引起肿瘤对EGFR-TKIs的耐药；④上皮来源肿瘤获得间质样表型（EMT现象）引起的EGFR-TKIs耐药^[3]^。Chung等^[4]^报道了1例NSCLC患者在厄洛替尼治疗出现进展后，复发灶穿刺样本证实上皮标记E-Cadherin表达阴性，高表达间质细胞标记Vimentin；并且在体外CL-387、785（EGFR-TKIs）肺癌耐药株HCC827/CLR中重复了EMT表型。Suda等^[5]^将EGFR-TKIs敏感的肺癌HCC4006细胞用低剂量厄洛替尼持续诱导得到耐药株HCC4006/ER，分析细胞表型发现E-Cadherin表达缺失，而高表达间质细胞标记Vimentin，证实EMT也是EGFR-TKIs获得性耐药机制之一；⑤耐药蛋白通过药物外排途径介导EGFR-TKIs耐药。Ozvegy-Laczka等^[6]^通过系列体外实验发现吉非替尼和多药转运蛋白ABCG2间存在极高的亲和力，可抑制其介导的药物外排，也提示ABCG2等多药转运蛋白通过促使TKIs外排介导EGFR-TKIs耐药的可能；⑥细胞凋亡缺陷引起EGFR-TKIs耐药。BIM是Bcl-2促凋亡家族成员之一，也在介导EGFR-TKIs诱导细胞凋亡过程中起着重要作用，Faber等^[7]^的研究发现，在BIM mRNA低表达或多态性缺失的情况下，肿瘤细胞可逃避EGFR-TKIs诱导的细胞凋亡。

## 2 EMT在NSCLC EGFR-TKIs耐药中的作用

EMT是一类生物学基本现象，即上皮细胞转化为间质表型细胞，目前研究表明，其在肿瘤进展的过程也发挥了一定的作用。EMT过程中，上皮细胞标志物表达下调，如N-Cadherin、E-Cadherin，而Vimentin、纤连蛋白等间质标志物表达上调。Ren等^[8]^开展了一项回顾性研究，纳入202例晚期NSCLC患者，比较EGFR-TKIs药物有效率和患者用药前EMT基线表型之间的相关性，他们发现，上皮表型多见于*EGFR*突变的患者，且与野生型相比，*EGFR*突变患者的预后更好。此外，肿瘤细胞表型并不是固定不变的，经过化疗或靶向等治疗后，起初的EMT表型或许会发生变化，例如烷化类化疗药如顺铂，就可能会诱导EMT^[9]^，继而产生吉非替尼耐药。在EGFR-TKIs耐药人群二次活检样本中，间质标志物表达增多，而上皮标志物表达阳性的患者，EGFR-TKIs药物治疗效果更好，即药物治疗有效率更高，无进展生存期更长。此外还有研究表明，NSCLC细胞系上皮表型恢复后，原本耐药的细胞重获对吉非替尼的敏感性。现从肿瘤微环境、信号通路、表观遗传3个方面总结目前对EMT效应获得EGFR-TKIs耐药机制的研究进展。

### 2.1 肿瘤微环境介导EMT效应获得EGFR-TKIs耐药

NSCLC肿瘤微环境由细胞成分和细胞外基质（excetral cellular matrix, ECM）组成，而细胞成分则由肿瘤细胞和周围肿瘤相关成纤维细胞（cancer-associated fibroblasts, CAFs）等其他间质细胞构成。CAFs可以通过释放TGF-β、细胞因子、趋化因子等诱导NSCLC细胞发生EMT，也可以释放基质金属蛋白酶（matrix metalloproteinases, MMPs）来调节细胞外基质，间接影响EMT过程^[10]^。Choe等^[11]^发现，PC9细胞在和CAFs共培养的过程中可发生EMT现象，七次跨膜蛋白SMO表达上调，Hedgehog信号通路显著激活，进而对厄洛替尼产生耐药。康小红等^[12]^发现，肿瘤微环境中的HGF可通过参与EMT进程介导了NSCLC对阿法替尼的原发耐药。Reckamp等^[13]^认为微环境中COX-2高表达可介导PGE2依赖的EMT，引起TKI耐药。而肿瘤细胞、巨噬细胞等细胞成分均可向微环境分泌COX-2等分子。

以上研究表明，肿瘤微环境中的间质细胞及其分泌的生物细胞因子参与EMT介导的TKIs耐药。

### 2.2 信号通路介导EMT效应获得EGFR-TKIs耐药

EMT的发生由细胞内信号转导通路精确调控，细胞外信号与细胞膜上相关受体结合，将信号传至细胞内，激活细胞内核转录因子，调控相关基因表达。而目前研究发现，在EMT介导NSCLC对EGFR-TKIs耐药的过程中，有多条信号通路被激活，包括SRC/FAK信号通路、Notch信号通路、Hedgehog信号通路以及IGF1R信号通路等，它们通过协同、拮抗相互交叉对NSCLC的EMT过程进行调节，进而诱导耐药产生。

#### 2.2.1 SRC信号通路介导EMT效应获得EGFR-TKIs耐药

Li等^[14]^研究，SRC抑制剂达沙替尼可通过抑制SRC/FAK信号通路对EMT相关的厄洛替尼耐药NSCLC患者起效，Wilson等^[15]^也通过体外实验证实达沙替尼可通过SRC/FAK信号通路降低EMT诱导的EGFR-TKIs耐药细胞的活性，且进一步筛选出该通路中和耐药关系较密切的几种磷酸激酶（EPHB1、FAK以及ACK-1）。此外，SRC还可通过调控下游的AKT和MEK诱导EMT相关的TKI耐药^[16]^。

#### 2.2.2 Notch信号通路介导EMT效应获得EGFR-TKIs耐药

近期研究表明，Notch通路也参与EMT相关的TKI耐药，和多种EMT转录、生长相关分子存在相互作用，包括Snail、Slug、TGF-β、FGF及PDGF等，Xie等^[17]^发现在吉非替尼耐药细胞株PC9/AB2中，Notch-1 mRNA及蛋白均呈高表达状态，但Notch家族其他成员（Notch-2-4）的表达量却无明显改变，Notch-1受体N1IC转入EGFR-TKIs敏感的PC9细胞后，可出现EMT现象且细胞对吉非替尼敏感性下降，反之，用siRNA沉默耐药细胞株的Notch-1后，可逆转上述现象，进一步研究其内在通路发现，p21 Waf1/Cip1可调节cyclin D1的表达，cyclin D1经β-catenin依赖的Wnt信号通路介导Notch-1 DNA组蛋白修饰来调节Notch1的表达，而Notch-1下游的靶基因*Hes-1*可抑制p21 Waf1/Cip1启动子的转录，在EMT相关耐药细胞中，Notch-1、cyclin D1表达上调，而p21 Waf1/Cip1则表达下调。

#### 2.2.3 Hedgehog信号通路介导EMT效应获得EGFR-TKIs耐药

Bai等^[18]^发现，NSCLC对EGFR-TKIs敏感的情况下，hedgehog（Hh）信号通路是沉默的，在耐药时则呈激活状态。外源性激活Hh信号通路可通过介导EMT诱导耐药。反之，GDC-0449抑制EMT表型细胞中Hh信号通路后，miR-200b和let-7c表达上调，细胞对药物的敏感性增加^[19]^。

#### 2.2.4 Hippo信号通路介导EMT效应获得EGFR-TKIs耐药

Hippo通路中TAZ表达上调，抑制靶基因（*CTGF*, *AXL*）的表达^[20]^以及TGFβ-miR200-MIG6等过程^[21]^，均和EMT相关的TKI耐药有关。

#### 2.2.5 IGF1R信号通路介导EMT效应获得EGFR-TKIs耐药

此外，还发现IGF1R激活ERK/AKT进而上调Snail的表达介导EMT引起EGFR-TKIs耐药，逆转EMT后能恢复耐药细胞系对EGFR-TKIs的敏感性^[22]^。

### 2.3 表观遗传调控EMT获得EGFR-TKIs耐药

肿瘤细胞EMT过程同样也受到表观遗传的复杂调控，包括DNA甲基化、组蛋白修饰以及非编码RNA等，而不同调控方式之间、甚至各种调控分子之间，都是有着相互关系的。

#### 2.3.1 DNA甲基化

研究^[23]^发现，内皮素（endoglin）启动子甲基化可沉默其表达，进而诱导EMT的发生。Shien等^[24]^研究发现，在发生EMT现象的耐药细胞株中，miRNA-200c存在DNA甲基化现象，且甲基化后其表达下调，而Hashida等^[25]^通过进一步的研究发现，miRNA-200c DNA甲基化发现的部位是在启动子区域。

#### 2.3.2 组蛋白翻译后修饰（乙酰化、甲基化）

Peinado等^[26]^实验证实，在小鼠细胞中Snail可募集HDACs，并与HDACs蛋白质N末端的SNAG结构域结合，从而对CDH1启动子区的组蛋白进行修饰，并抑制其转录，此外，ZEB1也可协同组蛋白去乙酰化酶SIRT1使CDH1启动子区的H3组蛋白去乙酰化来抑制转录过程。Tang等^[27]^发现，profilin-2通过C端互作，可影响HDAC1核转位，减少其在Smad2和Smad3启动子区域的募集，进而激活TGF-β1/Smad信号通路，诱导EMT。Chen等^[28]^发现，H3R2me1被PRMT5甲基化后，可通过募集WDR5激活EMT相关基因的转录，且和H3K4甲基化有协同作用，而H4R3me2s被PRMT5甲基化后，相关基因座的转录则明显受到抑制。而去甲基化也在EMT过程中发挥了一定的作用，去甲基酶LSD1通过其胺氧化酶结构域，来催化甲基化组蛋白N末端的生物胺的氧化，进而抑制基因的表达。

#### 2.3.3 非编码RNA（miRNA, lncRNA）

Park等^[16]^发现，在NSCLC中，CRIPTO1在miR-205调控参与下可激活ZEB1，诱导EMT，继而产生EGFR-TKIs耐药现象。Kitamura等^[29]^研究发现，MiR-134/487b/655复合物可通过调节TGF诱导出EMT相关的EGFR-TKIs耐药。而MiR-154则可直接与3’-UTR结合下调*ZEB2*基因表达进而抑制EMT转化^[30]^。Lee等^[31]^也发现miR-147可通过逆转EMT来恢复细胞对EGFR-TKIs药物的敏感性。而Garofalo等^[32]^发现MET可通过miR-103、miR-203下调，miR-221、miR-222、miR-30b和miR-30c的上调诱导EMT以及吉非替尼耐药，另外研究发现miRNA-200可下调ZEB1/2的表达，且ZEB1/2也可反过来下调miRNA-200家族，在miRNA-200家族和ZEB1/2之间形成一个互反馈回路。

Pan等^[33]^研究发现，NSCLC细胞中，LncRNA BC087858可通过上调ZEB1和Snail的表达来调控EMT相关的非T790M突变EGFR-TKIs耐药。而Cheng等^[34]^将非T790M突变型吉非替尼耐药细胞株中的UCA1沉默后发现，细胞在EMT过程受限的同时，还重获了对吉非替尼的敏感性，由此他们推测，lncRNA UCA1也参与了EMT介导的EGFR-TKIs耐药过程。

## 3 结语

越来越多的研究表明，EMT与NSCLC获得EGFR-TKIs耐药之间存在紧密联系，肿瘤微环境中的间质细胞或细胞因子、异常信号通路以及组蛋白修饰、非编码RNA等表观遗传均在耐药相关的EMT表型变化中发挥作用。深入地探究肿瘤发生EMT的特征、功能改变及其机制，将为EGFR-TKIs耐药的研究提供新方向和新治疗靶点。
